# Optically modified second harmonic generation in silicon oxynitride thin films via local layer heating

**DOI:** 10.1038/s41598-023-35593-8

**Published:** 2023-05-29

**Authors:** Jakub Lukeš, Vít Kanclíř, Jan Václavík, Radek Melich, Ulrike Fuchs, Karel Žídek

**Affiliations:** 1 TOPTEC Research Center, Institute of Plasma Physics of the Czech Academy of Sciences, Za Slovankou 1782/3, 182 00 Prague, Czech Republic; 2 Technical University of Liberec, Faculty of Mechatronics, Informatics and Interdisciplinary Studies, Studentská 1402/2, 461 17 Liberec, Czech Republic; 3 Asphericon GmbH, Stockholmer Str. 9, 07747 Jena, Germany

**Keywords:** Nonlinear optics, Surfaces, interfaces and thin films, Nonlinear optics

## Abstract

Strong second harmonic generation (SHG) in silicon nitride has been extensively studied—among others, in terms of laser-induced SHG enhancement in Si_3_N_4_ waveguides. This enhancement has been ascribed to the all-optical poling induced by the coherent photogalvanic effect. Yet, an analogous process for Si_3_N_4_ thin films has not been reported. Our article reports on the observation of laser-induced threefold SHG enhancement in Si_3_N_4_ thin films. The observed enhancement has many features similar to all-optical poling, such as highly nonlinear power dependence, cumulative effect, or connection to the Si_3_N_4_–Si interface. However, identical experiments for low-oxygen silicon oxynitride thin films lead to complex behavior, including laser-induced SHG reduction. Following a thorough experimental study, including the effects of repetition rate or pulse length, the observed results were ascribed to heat-induced SHG variation. In addition to revealing a new mechanism of laser-induced SHG variation, our results also provide a means to identify this mechanism.

## Introduction

Silicon nitride (Si_3_N_4_), as well as silicon oxynitrides (SiO_x_N_y_), have attracted attention from many prospective applications in optics. These materials are used for optical coating as a means of creating layers with a graded refractive index^1^. Nevertheless, recently the research into silicon nitride has been motivated predominantly by its nonlinear optical properties, including strong second harmonic generation (SHG)^2^. These properties can be utilized in waveguide structures, photonic crystal nanocavities, plasmonic structures, or optical modulators^3–5^.

Many studies have investigated the nonlinear optical characteristics of Si_3_N_4_, focusing in particular on the source of effective SHG^4–13^. The studies reveal two possible sources of SHG: (i) SHG generation on the Si_3_N_4_–Si interface^6,11^, and (ii) bulk-like SHG, which features dipolar character^4,6,13^. The bulk SHG with dipolar character has been assigned to breaking the material symmetry via strain or local inhomogeneities in these articles^11,14^.

In recent years, a number of workgroups have reported on the strong laser-induced enhancement of SHG in silicon nitride waveguides^15–17^ and microresonators^18,19^. This enhancement has been attributed to the effect of all-optical poling, where the driving mechanism was the so-called coherent photogalvanic effect. This effect induces internal local electric fields in the material and, therefore, allows efficient frequency doubling in centrosymmetric materials via third-order nonlinearity (EFISH)^15,16^. The photogalvanic effect occurs when a sample is exposed to the fundamental laser beam and its second harmonic, which can either originate from an external source or be generated in the sample itself.

The optically-induced SHG variation has also been reported for oxidized Si surfaces. The variation has been ascribed to the multiphoton electron and hole injection across the Si-SiO_2_ interface. However, this time-dependent SHG is restricted only to oxide layers of less than 10 nm and disappears for thicker layers^20^.

For SiO_x_N_y_ optical thin films exceeding 10 nm in thickness, it has been accepted that SHG efficiency is determined by the deposition process and thin film structuring. For instance, SHG intensity has been shown to be promoted by a stoichiometry of Si_3_N_4_^7^, a targeted deposition of Si_3_N_4_ and SiO_x_N_y_ structures with enhanced residual stress^21^, or accumulated fixed charges on layer interfaces^21^. In contrast to the waveguides and microresonators, optically induced SHG enhancement in optical thin films has not been previously reported.

In this article, we report on our observations of optically-induced SHG variation in silicon nitride and oxynitride thin films on a silicon substrate. In particular, we observed a strong threefold SHG enhancement on Si_3_N_4_ layers. Some characteristics of the enhancement closely resemble the coherent photogalvanic effect, including highly nonlinear power dependence or the cumulative character of the SHG enhancement^15,16^. Our measurements also revealed that the SHG variation is not linked to any notable change in the layer refractive index or chemical composition.

However, as our study extended to silicon oxynitride layers, we noted a more complex behavior depending on the layer stoichiometry. In some cases, the illumination by IR femtosecond pulses actually reduced the SHG intensity. The observed behavior was incompatible with the coherent photogalvanic effect.

Therefore, we carried out a set of experiments testing the presence of heat-induced changes in the sample, such as the effect of irradiation laser repetition rate, pulse length variation or ex-situ sample annealing. The acquired experimental results fully confirmed our hypothesis. Our results show that the all-optical SHG enhancement in silicon nitride and oxynitride can originate not only from optical poling but also from heat-induced material changes. The SHG variation can, therefore, be a complex process originating from an interplay of multiple phenomena.

## Results

### SHG variation in SiO_x_N_y_ thin films

As the first step, we studied laser-induced SHG enhancement in Si_3_N_4_ thin films deposited via dual ion beam sputtering on the Si substrate^22^. Throughout the study, we used p-polarized IR pulses at 1028 nm both for the sample irradiation and SHG measurement; the incident angle of the IR beam was 70 deg and we measured p-polarized SHG—see “Methods” for details^6^.

**Figure 1:**
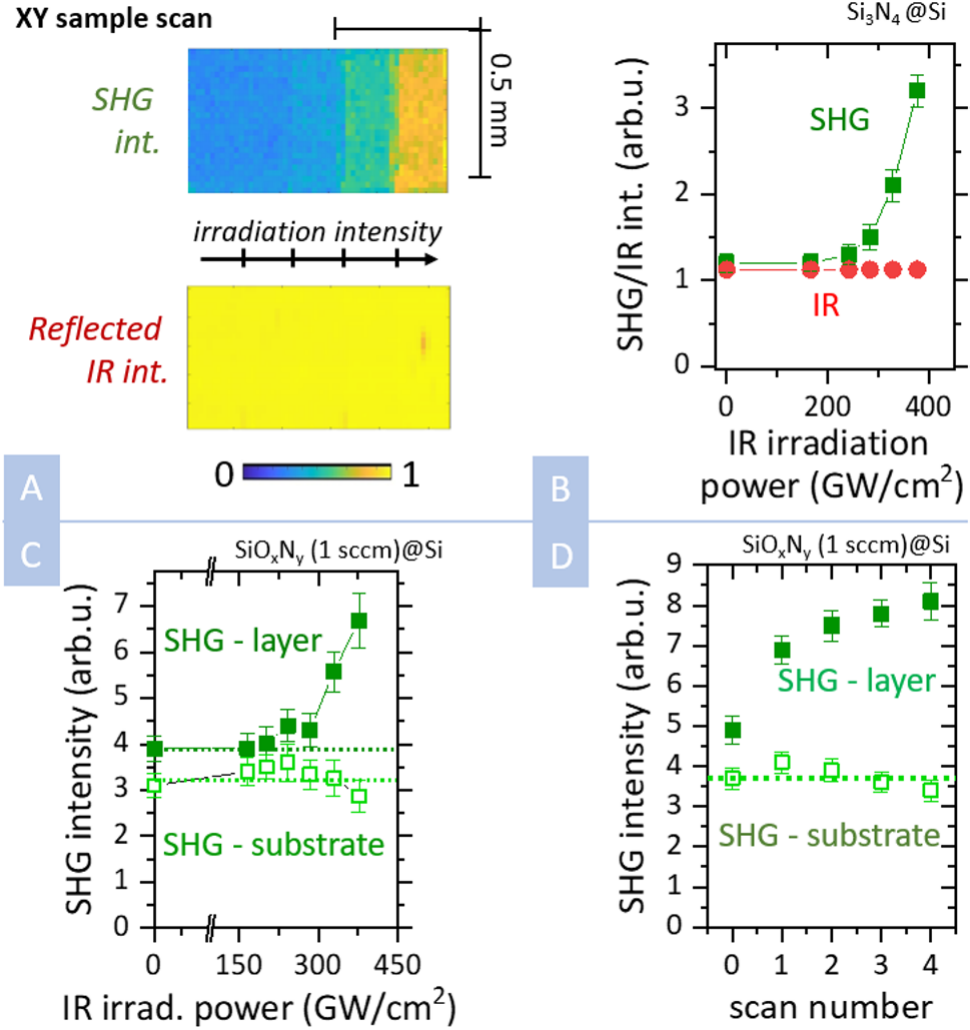
Laser-induced SHG enhancement (**A**) XY spatial scan of a sample—measured SHG and reflected IR intensities on Si_3_N_4_ layer previously illuminated in segments with a laser intensity increasing from left to right. (**B**) Mean SHG (green squares) and reflected IR (red circles) intensity after the previous illumination for a set of irradiation intensities (peak powers). Data measured for Si_3_N_4_ layer (0 sccm, Si substrate, 1500 nm thickness). The “zero” irradiation intensity corresponds to an unexposed layer. (**C**) SHG enhancement measured on SiO_x_N_y_ layer (1 sccm, Si substrate, 1200 nm)—solid squares. Compared to SHG intensity on the adjacent Si substrate—open squares. (**D**) SHG enhancement dependence on the number of illumination scans over the same segment. Measured on SiO_x_N_y_ layer (1 sccm, Si substrate, 1200 nm)—solid squares. Compared to SHG intensity on the adjacent Si substrate—open squares.

Laser-induced SHG variation measurements were implemented by placing a sample on an XY stage, which allowed us to irradiate the sample point-by-point and also to map the SHG intensity, as well as IR reflectivity. The laser-induced SH intensity variation was measured by illuminating several rectangular segments of the sample, using a different IR laser intensity for each segment—see Fig. 1A. Subsequently, we performed one overall XY scan at a low laser intensity, which was used to record the SHG and reflected IR intensity—see Fig. 1A.

We observed significantly enhanced SHG in the regions previously illuminated by high-intensity IR pulses—see Fig. 1A. The dependence of SHG enhancement on irradiation power in Fig. 1B followed the I^6^, which had been previously reported also for optical poling via the coherent photogalvanic effect^15,16^. The SHG enhancement was homogeneous across the illuminated area, which implies that it was not connected to any local contamination of the sample. The enhancement also did not alter the dependence of the SHG on the polarization of the input beam—see Supplementary Fig. 5 in Supplementary information.

In parallel to the SHG measurement, we also measured the IR reflectivity of the illuminated area (see Fig. 1A,B), which remained constant within the relative statistical error of 0.5%. Since we measured the p-polarization reflectance in the proximity of the Brewster angle, we can infer that there cannot be any major change in the refractive index of the layer or substrate. Analogous SHG enhancement was also observed for oxynitride thin films deposited on a silicon substrate with 1 sccm oxygen flow—see Fig. 1C. We denote silicon oxynitride layers by the flow of oxygen used during the layer deposition. While ϕ(O_2_) = 0 sccm corresponds to the pure Si_3_N_4_, the flow of ϕ(O_2_) = 3 sccm leads to the formation of nearly SiO_2_ layers^23^. The estimate of stoichiometric factors for each sample is provided in Supplementary information, Sect. 1.

We also studied the case where a segment of a sample is irradiated multiple times—see Fig. 1D. We noticed that the effect is cumulative, i.e., SHG enhancement grows with a higher number of scans but it shows signs of saturation after a few repetitions. A similar effect could also be observed for irradiation by the same intensity level for time intervals ranging from 0.5 to 10 s. We observed that a 10-s irradiation leads to a 10–20% increase in SHG enhancement compared to a 0.5-s irradiation (see Supplementary information for details). Nevertheless, the cumulative effect of multiple scans is much more pronounced.

**Figure 2:**
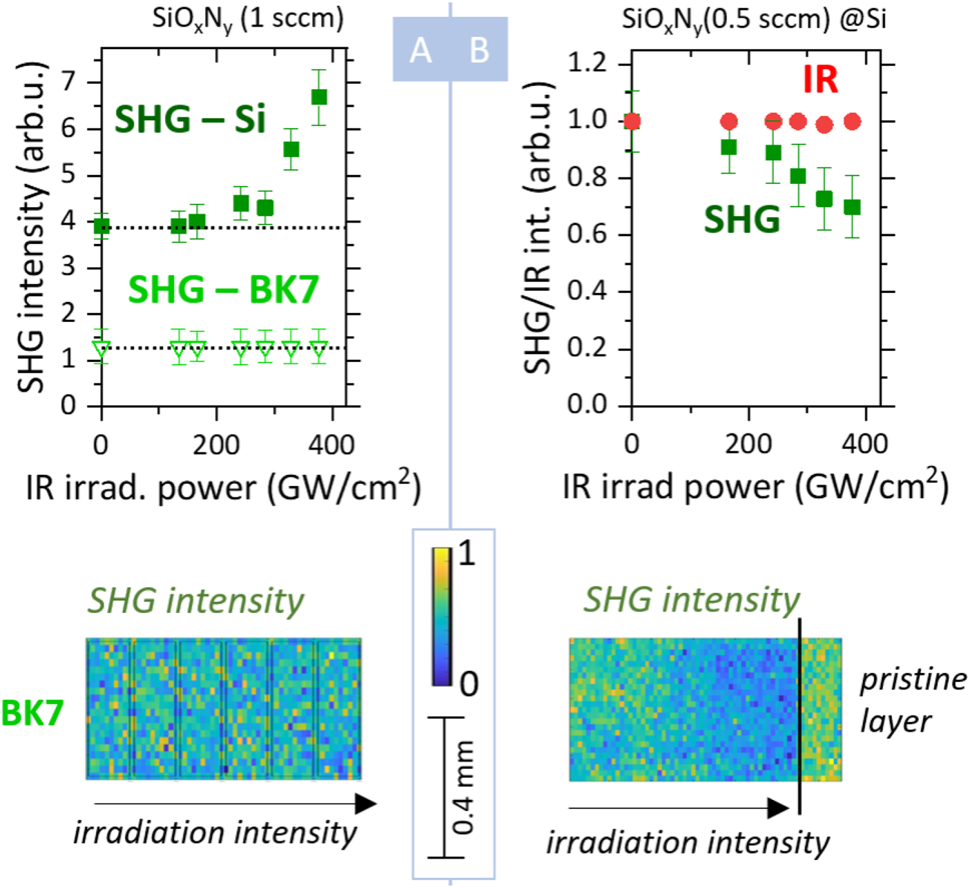
(**A**) Comparison between SHG enhancement of SiO_x_N_y_ thin layer (1 sccm, 1200 nm) deposited on Si substrate (solid squares) and same layer deposited on BK7 substrate (open triangles). Lower part: SHG intensity map—XY scan of the illuminated layer on BK7. (**B**) SHG intensity (green squares) and reflected IR intensity (red circles) after laser illumination on SiO_x_N_y_ layer (0.5 sccm, 1200 nm, Si substrate)—dependence on the illumination power. Lower part: SHG intensity map—XY scan of the illuminated layer. The pristine (reference) area was not illuminated prior to the measurement.

Interestingly, we observed the laser-induced SHG variation only for the layers on the Si substrate, while the same layers deposited on the BK7 substrate did not show any sign of enhancement—see Fig. 2A. We confirmed this behavior for a variety of SiO_x_N_y_ thin films deposited within the same batch, which differed only by their substrate. Therefore, in the following text, we restricted ourselves to layers deposited on the Si substrate. We discuss the implications of the difference between the substrates in the “Discussion” section.

To exclude the possibility that the laser-induced changes occurred in the substrate only, we carried out measurements close to the layer edge, where we irradiated the layer and the bare substrate within a single experiment. The Si substrate without a layer showed only minor SHG changes within the statistical error of the measurement—see Fig. 1C,D. We also verified that the amplitude of the IR laser electric field was comparable for the bare substrate and the substrate below the layer. Therefore, the absence of SHG enhancement on the bare substrate shows that the SHG variation originated in the layer, and it requires the presence of the Si-layer interface.

The observed properties of SHG enhancement presented in Figs. 1 and 2A closely resemble the coherent photogalvanic effect reported previously on Si_3_N_4_ waveguides. The similarities include the I^6^ nonlinear behavior, cumulative SHG enhancement, SHG enhancement without apparent change in the linear optical response of the layer, and connection to the Si–SiO_x_N_y_ interface.

The photoinduced SHG variation became more complex for the low-oxygen oxynitride thin film samples. We observed that for SiO_x_N_y_ layers with an oxygen flow of 0.25–0.5 sccm, the SH intensity decreased with the increasing IR illumination intensity—see Fig. 2B. This behavior was persistent for different deposited samples.

The SHG reduction on the low-oxygen oxynitride thin films in Fig. 2B contradicted the interpretation via optical poling. The coherent photogalvanic effect is inherently phase-matched and always leads to SHG enhancement^15^. Moreover, previously reported methods of erasing the built-in internal electric field by illuminating the sample with an externally generated SH light have been proven not to affect the observed changes in our samples^15^.

### Heat-induced SHG variation

We propose a different phenomenon consistent with the presented experimental data as the source of the SHG variation: heat-induced changes. By measuring the transmitted and reflected pulse energy, we verified that the vast majority of the pulse energy was absorbed within the layer and Si substrate. The linear absorption was dominated by the 1 mm thick Si substrate, while the losses within the layer are negligible—see Supplementary Information, Sect. 7. Nevertheless, the excitation intensities used considerably exceeded the linear regime^24^.

To evaluate the possibility of a heat-induced sample transformation, we carried out a simple calculation, which provided us with a gross estimate of the temperature change induced by a single pulse—see Supplementary information, Sect. 4 for details. Depending on the absorption depth, we can estimate that a single pulse can increase the local temperature by tens of Kelvins at most but cannot by itself provide enough heat for layer restructuring. This implies that the potential heat-induced effect must be cumulative.

Therefore, we carried out sets of measurements where we varied the laser repetition rate. If a coherent photogalvanic effect or an analogous nonlinear process is responsible for the SHG enhancement, we should observe the same SHG enhancement when we keep a constant irradiation peak power and the same number of incident pulses on the spot. This would hold irrespective of the laser repetition rate. On the contrary, the heat-induced changes in a sample should strongly depend on the laser repetition rate, even if the total number of incident pulses is the same. This is because the long delay between the pulses allows for better heat dissipation between the pulses.

**Figure 3:**
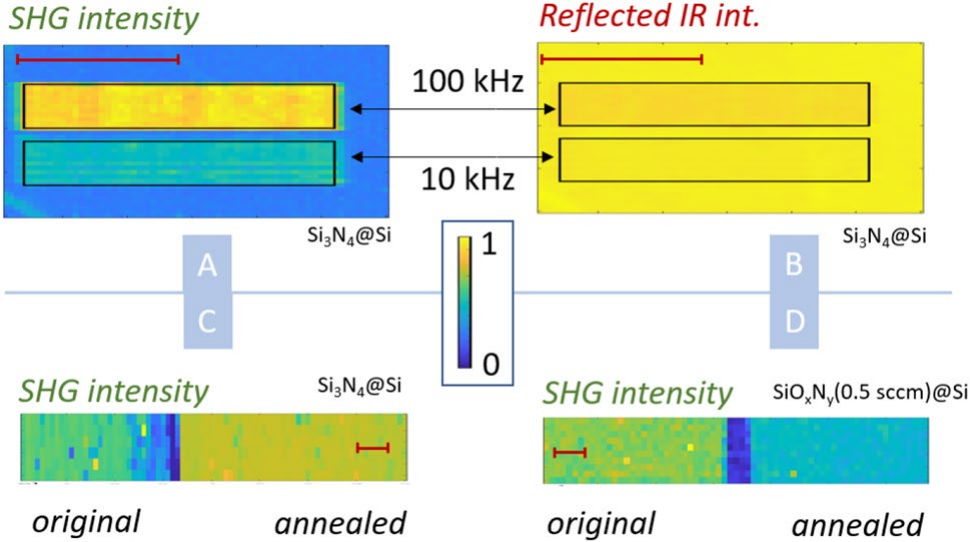
(**A**) XY scan of SHG intensity of Si_3_N_4_ layer (0 sccm, 1500 nm, Si substrate) irradiated by two laser repetition rates: 100 kHz for 2 s (upper rectangular area) and 10 kHz for 20 s (bottom rectangular area), irradiation laser intensity 230 GW/cm^2^. (**B**) XY scan of reflected IR intensity—same conditions and sample as in panel (**A**). (**C**) Thermal enhancement of SHG for Si_3_N_4_ layer via ex-situ annealing: XY scan over two segments of the same sample—LHS: without heat treatment; RHS: 400 °C annealed. (**D**) Thermal enhancement of SHG for SiO_x_N_y_ with sccm 0.5 via ex-situ annealing (LHS without heat treatment, RHS 400 °C annealed). The red scale bars correspond to 0.5 mm for all panels.

By carrying out the SHG enhancement measurement for 100 kHz and 10 kHz repetition rates where we maintained the same number of incident pulses, we could see that the enhancement is highly dependent on the laser repetition rate—see Fig. 3A,B. This result indicates that we observe heat-induced changes in the Si–SiO_x_N_y_ interface.

To confirm this conclusion, we conducted an experiment with ex-situ sample annealing. We cut an Si substrate with an Si_3_N_4_ layer into two parts. One of those parts was uniformly heated to 400 °C for 30 min and then left to cool down at room temperature. Both parts of the sample were then placed into the setup and scanned as a single measurement. The results can be seen in Fig. 3C, where the annealed part of the sample is shown on the right-hand side, while the left-hand side is the part without thermal treatment. The average measured intensity of the SH of the annealed part is approximately 20% higher compared to the non-annealed part. At the same time, changes in reflected IR intensity are subtle.

Moreover, we conducted the same experiment on the SiO_x_N_y_ sample (0.5 sccm) for which we had previously observed a decrease in SHG due to irradiation with a laser beam—see Fig. 2B. In line with our expectations, here, the annealing had led to the reduction of SHG average intensity on the annealed part by approximately 30% in comparison to the part which had not been heat treated—see Fig. 3D.

**Figure 4:**
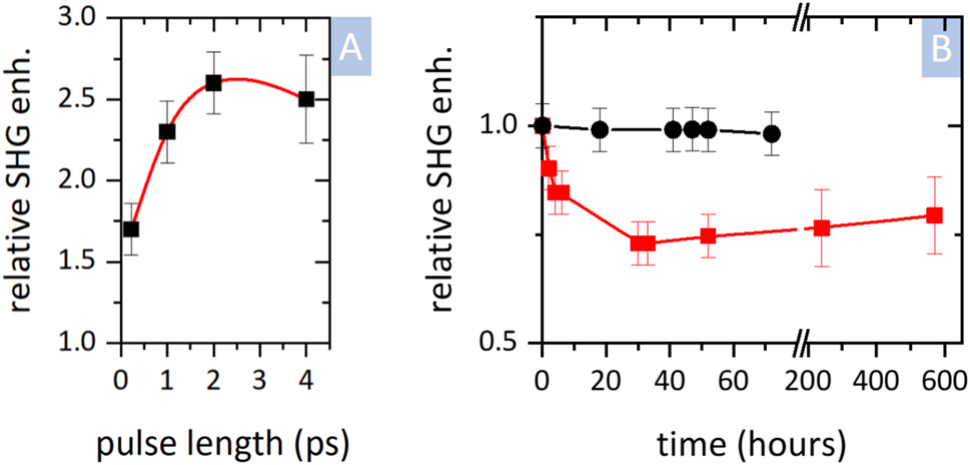
(**A**) Relative SHG intensity enhancement of Si_3_N_4_ layer (0 sccm, 1500 nm, Si substrate) irradiated by a varying pulse length for a constant total pulse energy (46 mJ/cm^2^). Value 1 corresponds to SHG intensity without enhancement. The red line serves only as a guide for the eye. (**B**) Relaxation of the SHG enhancement after IR irradiation for Si_3_N_4_ layer. Red symbols: Si_3_N_4_ layer (0 sccm, 1500 nm, Si substrate). Black symbols: Si_3_N_4_ layer (0 sccm, 600 nm, Si substrate); The samples were irradiated by pulse energy 46 mJ/cm^2^ (peak power 200 GW/cm^2^) for standard conditions. Value 1 corresponds to the initially measured SHG intensity immediately after irradiation. The lines serves only as a guide for the eye.

To elucidate the mechanism of nonlinear light absorption, we studied the effect of laser peak power on SHG variation. Namely, we tuned the incident pulse length between 225 fs and 4 ps and measured SHG enhancement in Si_3_N_4_, while all other properties, including energy per pulse and repetition rate, were kept constant. We observed that the relative SHG enhancement increased by 50% by prolonging the pulse length from 225 fs up to 2 ps—see Fig. 4A. Although we discuss these results in the following section, it should be noted here that this observation provides strong evidence against optical poling being responsible for SHG enhancement in our samples. The asymmetric photogalvanic current induced during the optical poling is driven by a nonlinear interaction of charge carriers with electric field, which is proportional to the pulse peak power^15^. The peak power was almost ninefold decreased in the 2-ps pulses, yet the SHG enhancement increased.

Finally, we also studied the relaxation of the SHG variation in time. In particular, we measured Si_3_N_4_ layers on an Si substrate, which underwent an IR irradiation leading to an SHG enhancement. We used samples with different layer thicknesses and irradiation intensities. The enhancement scale was measured immediately following irradiation via multiple scans to verify that the scanning itself did not alter the measured value. Subsequently, the samples were repeatedly measured for several days—see Fig. 4B. Depending on the conditions, i.e., layer thickness and irradiation power, we observed a different degree of SHG relaxation. For the samples with a weakly enhanced SHG, the SHG relaxation was negligible–see black symbols in Fig. 4B (example for 10% SHG enhancement). On the contrary, for the strongly enhanced SHG, the enhancement factor decreased up to 75% of the original value—see red symbols in Fig. 4B (example for 60% SHG enhancement). Interestingly, such enhancement relaxation on the scale of several days is similar to the relaxation of SHG enhancement observed for optical poling^25^.

## Discussion

In light of our results, we can safely ascribe the observed laser-induced SHG enhancement/reduction to localized layer heating. From the observed absence of the SHG enhancement for the BK7 substrate, we can conclude that the induced heat must be generated either on the layer-Si interface or in the adjacent Si substrate itself. This interpretation is also consistent with other experiments carried on the same samples, such a pump-probe study of the initial dynamics on the picosecond timescale—see Supplementary information, Sect. 5 for the results. Nevertheless, on longer timescales, heat conduction would lead to temperature uniformity throughout the whole layer depth, which we have verified by a simple finite-element method simulation.

A highly nonlinear character of the SHG enhancement (I^6^ dependence) was combined with a more pronounced SHG enhancement for an increased pulse length. Such behavior is consistent with the observation of free-carrier absorption, i.e., induced absorption on excited charge carriers, which is followed for high laser intensities by impact ionization, where the excess energy of excited charge carriers can lead to the excitation of new ones. The intraband free-carrier absorption of excited carriers enables a rapid transformation of excess energy into heat via interaction with optical phonons. Nevertheless, our experimental results do not allow us to discern the free-carrier absorption at the layer-substrate interface from the same process in the adjacent substrate.

We can now turn to the identification of the mechanism behind the SHG enhancement itself. In the published literature, SHG in Si_3_N_4_–Si structures is commonly attributed to SHG from the Si–Si3N4 interface and dipole generation from the Si_3_N_4_ bulk. Our results alone cannot resolve the difference between the sources and provide us with direct evidence about the actual physical mechanism of heat-induced SHG enhancement. Yet, we can discuss the consistency of various scenarios with the experimental results.

We propose two viable scenarios of SHG variation: (i) formation of a new heat-induced sublayer at the layer-substrate interface leading to the change in the interface SHG, (ii) heat-induced restructuring of the layer bulk alternating the efficiency of the bulk SHG.

We have previously observed that for the studied Si_3_N_4_ layers deposited via IBS, the SHG efficiency scales with the layer thickness, and, also, its angular dependence suggests that the bulk-like SHG dominates for layers with a thickness exceeding 1 μm^6^. If the enhancement originated from the SHG on the layer-Si interface, we would expect to observe a lower SHG enhancement for higher Si_3_N_4_ layer thickness because the dominating bulk-like SHG would remain unchanged. However, this is contrary to the results, where we have observed analogous levels of SHG enhancements for layers with thicknesses varying more than threefold.

We can also discuss the results with respect to the IR reflectance of the samples, which showed only negligible changes following high-intensity IR irradiation. To do this, we created an optical model of the sample—see Supplementary Information, Sect. 5. The optical model studies the variation in the IR reflection of the sample induced by a change in the refractive index of the thin films. From the model, we can conclude that the observed subtle changes in IR reflectance put constraints on the two above-mentioned scenarios. The change in the refractive index can be very high (> 0.1) only for the formation of a very thin layer (< 10 nm). For the layer bulk modification (> 50 nm thick), the refractive index is expected not to vary by more than 0.01.

Heat can, in principle, induce the formation of a very thin sublayer at the layer-substrate interface via atom diffusion across the interface. However, the diffusion coefficients of Si and N in Si_3_N_4_ and the corresponding diffusion length during the short illumination period of 2 s would reach a notable effect only when the local temperature in the layer was significantly above 1700 °C^26^, i.e., exceeding the melting point of Si and approaching the melting point of Si_3_N_4_. This temperature level is not viable since we have observed that the layer can resist a significantly higher irradiation level and the related temperature without being destroyed. Diffusion of atoms (e.g., oxygen) across the layer-air interface cannot be responsible for the enhancement, as this process would be active for both Si and BK7 substrates.

Therefore, we propose heat-induced restructuring of the layer bulk as the most feasible explanation. We can speculate that the mechanical stress in the layer might be responsible for the SHG variation, as the stress: (i) highly affects SHG efficiency due to symmetry breaking^21^; (ii) leads to a low refractive index change^27^; (iii) its hysteresis is highly different for Si_3_N_4_ and SiO_x_N_y_ layers, as the character of the stress changes from compressive to tensile^28^. Our experiments with layer annealing at 400 °C, which is sufficient to change the stress in the layer^29^, led to SHG modification in line with laser-induced changes. Finally, the partial relaxation of the SHG enhancement might be explained by the relaxation of residual stress in the layers over the range of several days, which has been previously reported in the literature^30^.

It is worth stressing again that the discussion of the physical origin of the SHG enhancement is based only on indirect evidence, where we discuss the consistency of the proposed mechanisms with the observed results. While we can safely assign the SHG enhancement to heat induced in the samples by the focused laser beam, the actual heat-induced mechanisms cannot be confirmed or excluded with certainty.

In summary, we carried out a thorough investigation of light-induced SHG modification, which we observed, unlike other reports, on thin-film samples. While the nonlinear behavior and other aspects might suggest that we witnessed the coherent photogalvanic effect, we carried out a set of measurements that safely ascribed the SHG modification to localized heating of the layer. We propose that the heat-induced restructuring of the layer bulk is the most viable source of the observed SHG enhancement; however, we cannot exclude other options.

First of all, our results provide information on a new mechanism of laser-induced SHG variation in Si_3_N_4_ and SiO_x_N_y_ thin-film layers on a Si substrate. This mechanism is entirely different from the previously reported optical poling. Therefore, it is of great interest to distinguish the two effects in future experiments and to take SHG enhancement into account in layer characterization.

In particular, we propose measurements of SHG under various laser repetition rates and varying laser pulse length as a simple way to verify the presence of heat-induced modification. In general, for studying Si_3_N_4_ layers, it is highly beneficial to use a reduced laser repetition rate as a simple means of minimizing laser-induced layer modification^6^.

## Methods

### Second harmonic generation setup

The SHG setup used in this study was described in detail in Ref. 6. Amplified Yb:YAG fs pulses (4 µJ/pulse) at 1028 nm were directed into the SHG setup, where their intensity was modulated to the desired level using a λ/2 waveplate and a polarizing cube beam splitter. The IR intensity level at the sample was calibrated by a thermal power meter (thermopile). The IR pulses were focused onto the sample into a spot 20 µm in diameter, which was determined using the knife-edge technique. The focused IR beam was used to generate SHG in the reflective geometry.

Throughout the article, we used p-polarization IR light, incident angle 70 deg, and we detected p-polarization SHG radiation. Using a series of dichroic optics and color filters, we simultaneously detected the intensity of the reflected IR light and the generated SH radiation. Unless stated otherwise, the experiments were carried out at the laser repetition rate of 100 kHz.

Unless stated otherwise, the IR pulses were compressed into the pulse length of 225 fs. By using the grating compressor integrated in the laser, we were able to increase the pulse length up to several picoseconds by inducing a chirp in the pulses. The chirped pulse length was determined based on the calibration provided by the manufacturer (Light Conversion). The compression of pulses was optimized by supercontinuum generation in sapphire.

### Sample preparation

Sample preparation was carried out using the dual ion beam sputtering described in detail in Ref.^6^. A beam of Ar^+^ ions (beam current 108 mA, beam voltage 600 V) sputtered Si atoms from a target onto substrates (Silicon or BK7), where the deposited atoms interacted with nitrogen and oxygen ions generated by an assistance ion beam (emission current 0.6 A, discharge voltage 70 V). By changing the ratio between the oxygen and nitrogen ion flux, we were able to vary the stoichiometry of the layers from the pure Si_3_N_4_ (ϕ(O_2_) = 0 sccm) through SiO_x_N_y_ to nearly SiO_2_layers (ϕ(O_2_) = 3 sccm). The estimate of stoichiometric factors is provided in Supplementary information (Sect. 1). We carried out a detailed study of the linear optical properties of the layers in Ref.^23^. The thickness of the layers varied between 300 and 3500 nm.

The deposited layers, when being left at room temperature, were observed to be stable in terms of their optical properties (transmittance and reflectance) on the scale of years.

### Laser-induced SHG variation

Laser-induced SHG variation measurements were implemented by placing a sample on an XY stage, which allowed us to scan the sample and irradiate it point by point. The laser-induced SH intensity variation was measured by illuminating several rectangular segments of the sample, using a different IR laser intensity for each segment—see Fig. 1A.

During the irradiation, the segment was scanned spot-by-spot by the IR laser (1028 nm), irradiating each spot for 2 s unless stated otherwise. The irradiation intensity ranged from 170 to 430 GW/cm^2^, corresponding to 35–100 mJ/cm^2^/pulse. Subsequently, we performed one overall XY scan recording the SH and reflected IR intensity—see Fig. 1A. The overall XY scan covered all previously irradiated segments and an adjacent reference area without prior irradiation. During the overall scan, the intensity of the IR beam was kept at a constant low level (170 GW/cm^2^, 35 mJ/cm^2^/pulse) to avoid additional sample changes. The data gained from the overall scan were then evaluated independently for each segment by calculating the average intensity and the standard deviation as an error estimation—see Fig. 1B.

The long-term reproducibility of the measurements was assured by using a laser with a stabilized output power. The pulse compression stability was verified by a supercontinuum generation in a sapphire plate.

## Supplementary Information

Supplementary Information.

## Data Availability

Data underlying the results presented in this paper are not publicly available but may be obtained from the corresponding author Karel Žídek upon reasonable request.
