# Supplementary material for: Optically modified second harmonic generation in silicon oxynitride thin films via local layer heating
Source: Sci Rep. 2023 May 29;13:8658. doi: 10.1038/s41598-023-35593-8 (PMC10227052; doi:10.1038/s41598-023-35593-8)
Supplement: Supplementary file 1 — Supplementary Information. [file 41598_2023_35593_MOESM1_ESM.pdf]

Supplementary information

Optically enhanced second harmonic generation in silicon oxynitride thin films via local layer heating

Jakub Lukeš,[1,2] Vít Kanclíř,[1,2] Jan Václavík,[1] Radek Melich,[1] Ulrike Fuchs,[3] Karel Žídek[1,*]

[1]Regional Center for Special Optics and Optoelectronic Systems TOPTEC, Institute of Plasma Physics of the Czech Academy of Sciences v.v.i., Za Slovankou 1782/3, 182 00 Prague 8, Czech Republic
[2]Technical University in Liberec, Faculty of Mechatronics, Informatics and Interdisciplinary Studies, Studentská 1402/2, 461 17 Liberec, Czech Republic
[3] Asphericon GmbH, Stockholmer Str. 9, 07747 Jena, Germany.
**Corresponding author: zidek@ipp.cas.cz*

1. Stoichiometry of the layers

Based on Bruggeman's effective-medium approximation, we were able to transpose the measured refractive index of the layers from Ref. [S1] into the estimate of their stoichiometry. In other words, we can connect the flow of oxygen used during the layer deposition with the layer stoichiometry. The Bruggeman's effective-medium approximation is commonly used for silicon oxynitride thin films. [S2]

Table S1. Estimated stoichiometry of the deposited layers based on Bruggeman's effective-medium approximation.

Φ O_2 (sccm)	Stoichiometry SiO_xN_y		
	x	y	
0	0	1.33	Si_3N_4
0.25	0.26	1.16	$SiO_{0.26}N_{1.16}$
0.5	0.52	0.99	$SiO_{0.52}N_{0.99}$
0.75	0.74	0.84	$SiO_{0.74}N_{0.84}$
1	0.94	0.74	$SiO_{0.94}N_{0.71}$
1.25	1.14	0.57	$SiO_{1.14}N_{0.57}$
1.5	1.32	0.45	$SiO_{1.32}N_{0.45}$
1.75	1.48	0.35	$SiO_{1.48}N_{0.35}$
2	1.62	0.25	$SiO_{1.62}N_{0.25}$
2.5	1.84	0.11	$SiO_{1.84}N_{0.11}$
3	2	0	SiO_2

2. Additional characterization of the layers

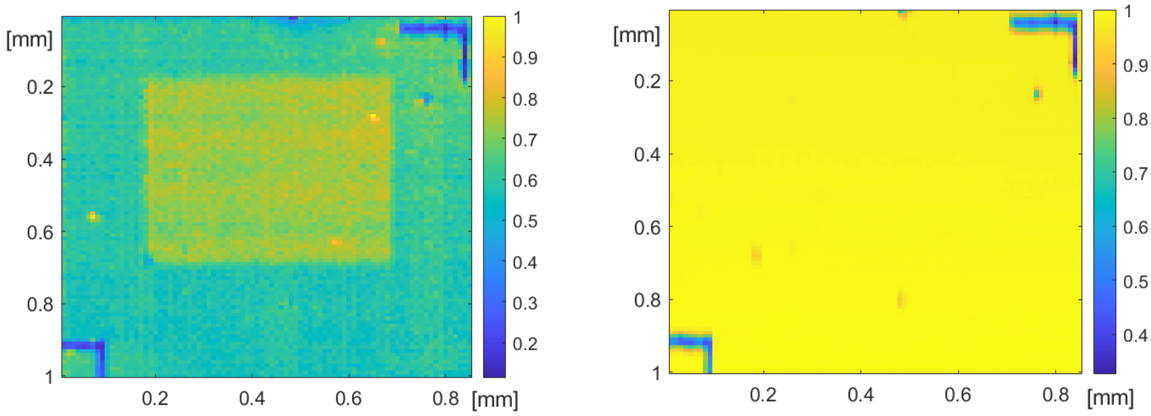

Figure 1. SHG intensity (left-hand side) and reflected IR intensity (right-hand side) XY scan of Si_3N_4 layer at Si substrate after irradiation of a central part by IR laser – see a brighter area in the SHG intensity. Note markers in the left lower and right upper corners.

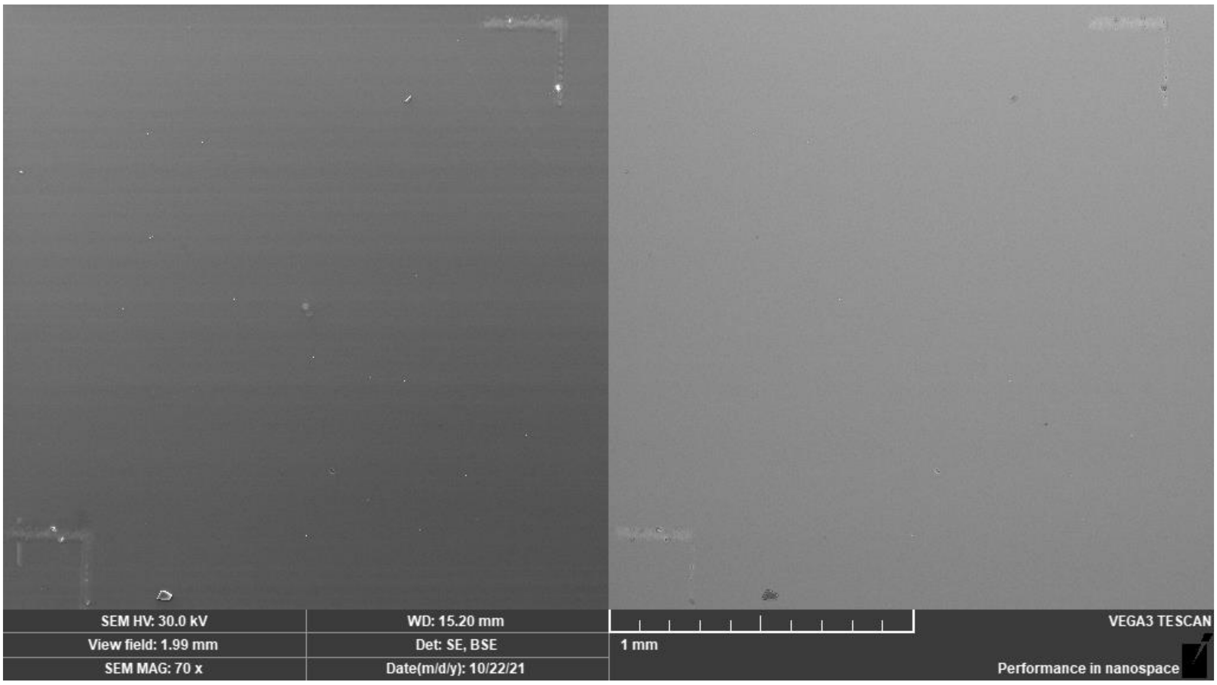

Figure 2. A scanning electron microscope image of an area of a sample (Si_3N_4 layer at Si substrate after irradiation) showed in Figure 1 with markers. Images are formed via secondary electrons (left-hand side) and back-scattered electrons (right-hand side). Electron microscope VEGA3 Tescan, accelerating voltage 30 kV.

3. Additional data for SHG enhancement

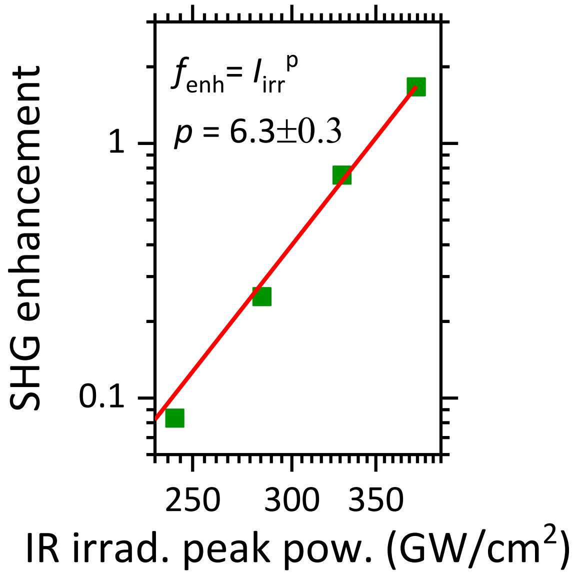

Figure 3. SHG enhancement dependence on IR irradiation peak power. Log-log scales are used to demonstrate a highly nonlinear rise as I_{Irr}^{6}.

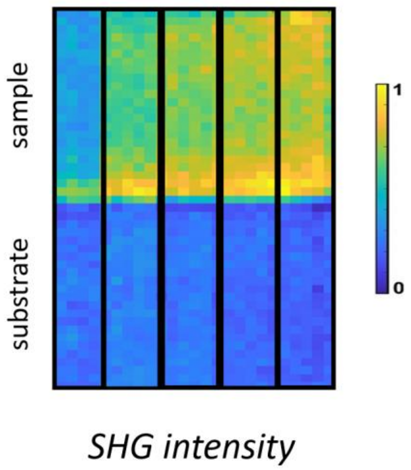

Figure 4. SHG intensity XY scan of the multiply irradiated areas after laser illumination on SiO_xN_y layer (1 sccm, 1200 nm, Si substrate). Segments correspond from left to right to 0, 1, 2, 3, and 4-fold irradiation. Data are processed in Figure 1D in the main article.

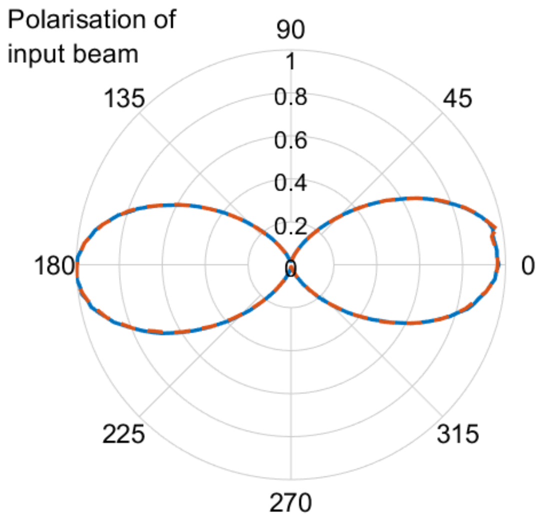

Figure 5. Normalized intensity of the p-polarized SH as the function of the incident beam polarization. The values of 0 deg. and 180 deg. correspond to the incident p-polarization, i.e., "pP" configuration. The curves were measured for pristine (solid orange line) and irradiated layer (dashed blue line). The two curves perfectly overlap.

4. Single-pulse absorption

Supposedly that a single pulse with energy E = 50 mJ/cm^2 is absorbed within the thickness *d*, then we should observe in this region the temperature difference, which can be estimated as:

$$\Delta T = \frac{E\varrho}{dC}, \qquad \text{(S1)}$$

where *C* stands for heat capacity, ϱ is the material density, *E* is the pulse energy, and *d* is the absorption depth. Since this effect is connected with the Si substrate, we implemented Si material constants C = 0.71 J/g/K, ϱ = 2.33 g/cm^3. A linear absorption coefficient of the 1028 nm light in Si takes place over a very long distance of 300 μm, for which the temperature change induced by a single pulse would be negligible (1 K). Nevertheless, even an extremely strong absorption over the thickness of 10 μm would cause the single pulse to elevate the temperature by about 30 K. Even though this estimate is gross, it is clear that a single pulse itself cannot induce the change in temperature that would affect the layer properties.

5. Ultrafast transient reflectivity – signatures of heat absorbance

To elucidate the initial charge carrier and phonon dynamics, we measured the transient reflectivity of the layers on the picosecond timescale. While the detailed description of this experiment is beyond the scope of this article, we will focus solely on the signatures of the heat absorbance in the sample.

The presented data were acquired in a single-color pump-probe experiment in reflective geometry; excitation wavelength 1028 nm, probe wavelength 514 nm (both 225 fs, 100 kHz, Pharos laser system). Figure 6 shows the measured transient reflectivity signal for the sample of SiO_xN_y (0.75 sccm, layer thickness 300 nm) on Si substrate. The inset in the upper right corner depicts oscillatory

features of the signal, which were extracted as the residuals from fitting the curve with a three-exponential function.

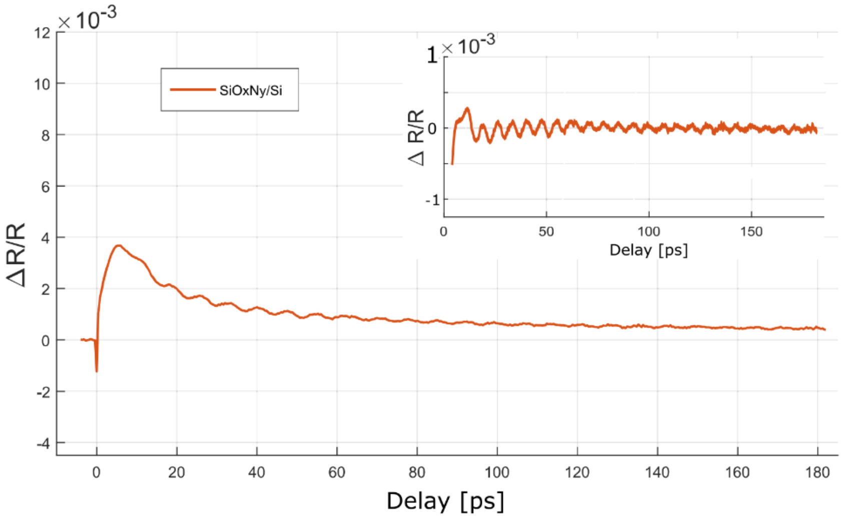

Figure 6. Transient reflectivity signal for SiO_xN_y layer (0.75 sccm, layer thickness 300 nm) on Si substrate. Excitation wavelength: 1028 nm; probe wavelength: 514 nm; rep. rate 100 kHz; excitation incident energy density: 70 μJ/cm^2/pulse. Inset: Oscillatory part of the transient reflectivity signal – see text for details.

From extensive measurements (data not shown here), we can safely assign the observed oscillatory signal (see Figure 6 inset) to the so-called Brillouin oscillation. The Brillouin oscillations arise due to a strain wave (acoustic shock wave) propagating through the Si substrate. The strain wave modifies the local refractive index of the material a causes the change in the total sample reflectance. We can use the data to calculate the longitudinal speed of the sound in Si: 8.48 nm/ps. [S3]

The strain wave is typically generated due to the localized heating of the material, i.e., it carries the information about the pulse absorption inducing the heat. The exact onset of the oscillations cannot be determined due to the overlapping signal from other processes. Nevertheless, the oscillatory features are clearly appearing within the first 8 ps. Therefore, we can safely claim that the dominating part of the absorbed heat is located in the Si substrate, or < 70 nm from the layer-substrate interface.

At the same time, the fact that we observe pronounced Brillouin oscillations implies that the induced strain wave is reasonably well localized. This is possible only in cases where the pulse is absorbed along a very short distance, which is smaller or comparable to the oscillation period (6 ps, corresponding to 50 nm distance). In the opposite case, the signal would be smeared out.

Therefore, transient reflectivity measurements revealed that the IR pulse in our sample generates a notable acoustic strain wave, which is initially well-localized (well below 1 μm). The wave is generated in the substrate itself or in direct proximity to the substrate -- less than 70 nm from the layer-substrate interface.

6. The model of IR reflectivity change

In our experiments, we measured the p-polarization reflectance at 70 deg. incident angle. This angle is close to the Brewster angle; therefore, any change in the refractive index in the system would lead to a major change in the reflectivity. For instance, the measurement in Figure 1, where we used a thick sample (1.5 μm layer thickness) with a very sensitive response, stayed constant within the relative error of the reflectance of 0.3%.

We created a model where we assume that a part of the Si_3N_4 layer adjacent to the substrate is transformed by the heat into a sub-layer with thickness d_{sub} a different refractive index Δn see Figure 7.

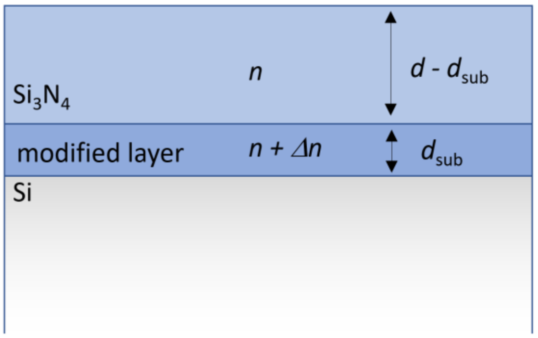

Figure 7. The model used to calculate the IR reflectivity change when a new sublayer is formed at the layer-substrate interface.

The resulting change in the IR reflectivity is shown in Figure 8, together with the IR reflectivity relative error of 0.5% as a solid black line. Therefore, the viable options supported by the stable IR reflection lie between the two black lines in the graph. Figure 8 implies that if there is a new sublayer to be induced by the IR light, it must be either very thin (< 15 nm) or the induced refractive index change must be extremely small (<0.02). In the opposite cases, the SHG enhancement would be accompanied by a major change in IR reflectivity.

It is possible to argue that the heat might induce both changes in the optical thickness and refractive index, which might cancel out and cause the reflectance at a single wavelength seemingly not to change. However, we point out that subtle changes in IR reflectance were consistently observed for various samples with different thicknesses, and it is highly unlikely that this cancellation would occur for all the variety of samples.

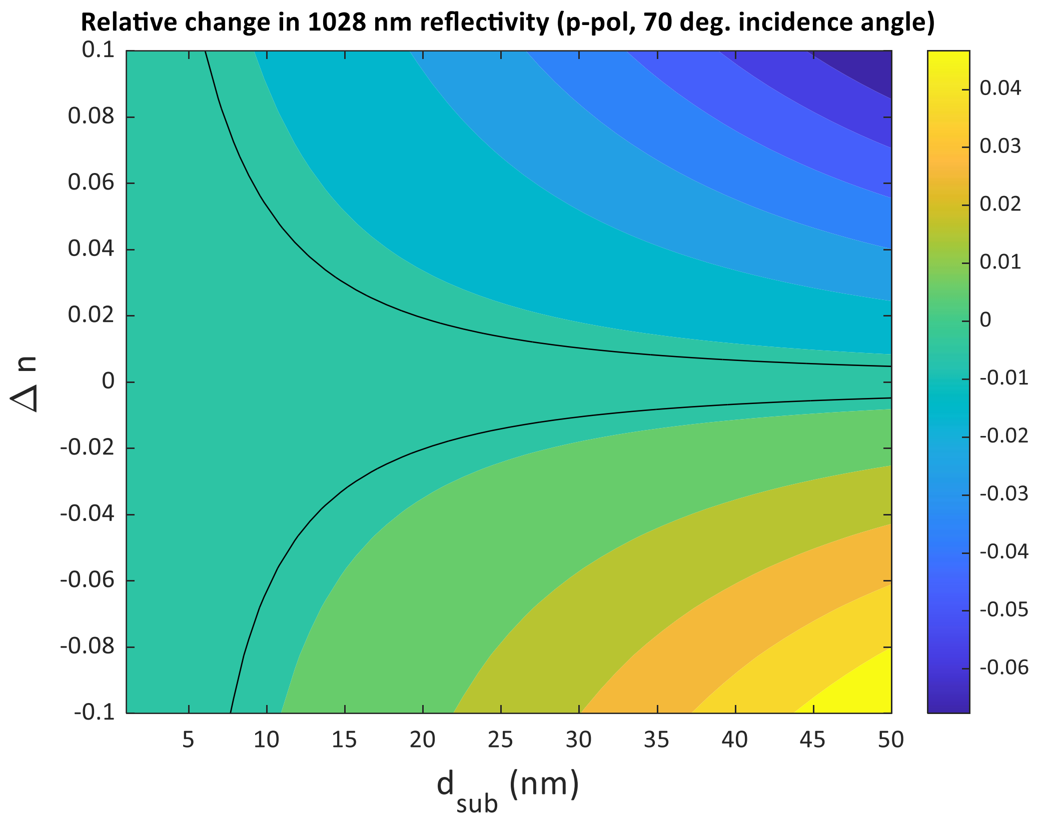

Figure 8. The relative change in reflectivity at 1028nm for p-polarization, 70 deg incidence angle for the layer and sub-layer system in Figure 7. The graph shows the dependence of the reflectivity change on sublayer thickness d_{sub} and refractive index change in the sub-layer Δn. Black lines denote the borders set by the experimental error of IR reflectance measurement.

7. Effect of exposition time on SHG enhancement

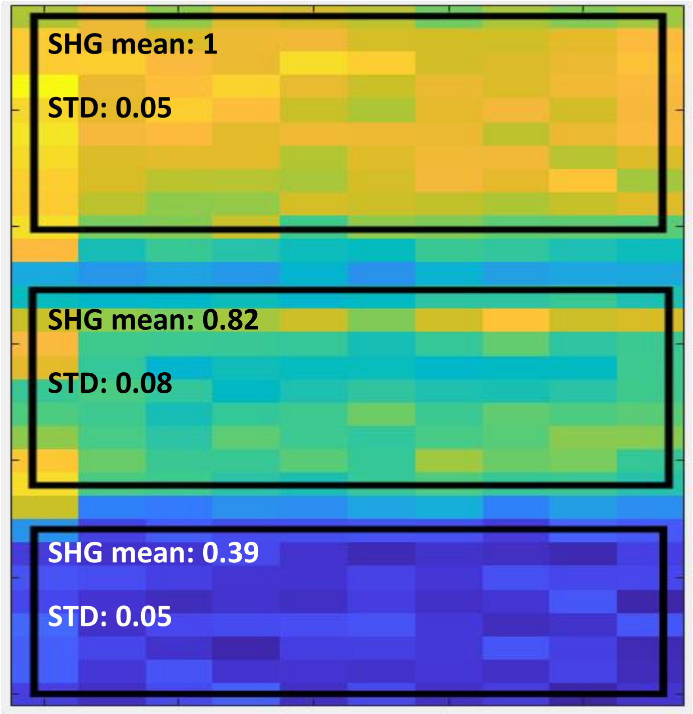

Figure 9. Two areas on Si_3N_4 layer deposited on Si were pre-irradiated with the same IR intensity but with different exposition times and then scanned with one overall scan. Mean values of SHG were normalized and in arbitrary units. Top area – 10 s IR pre-irradiation time; middle area – 0.5 s IR pre-irradiation time; bottom area – reference (without IR pre-irradiation)

8. Absorbance of Si_3N_4 layers

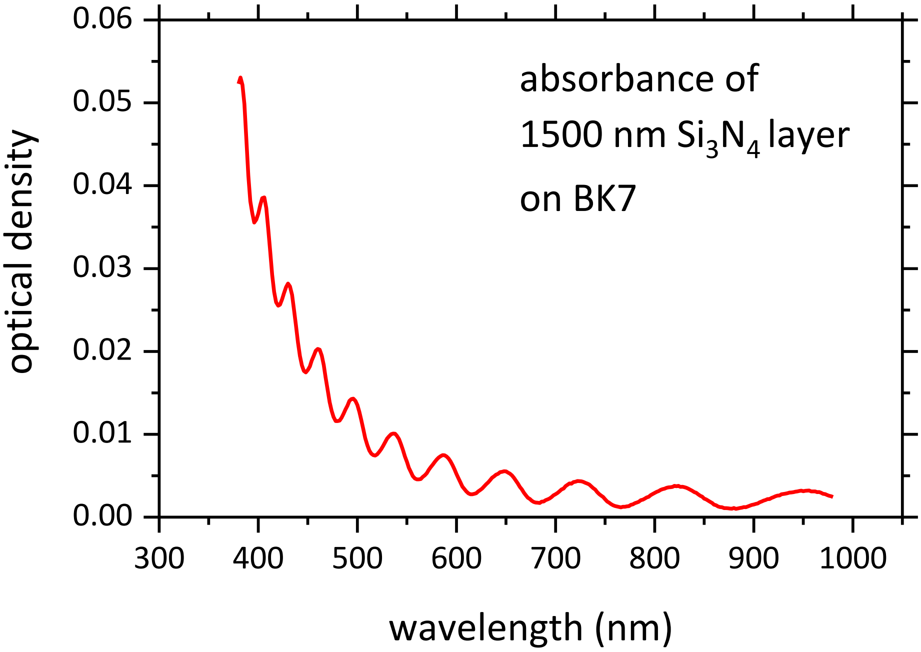

Figure 10. The optical density of Si_3N_4 layer deposited on the BK7 substrate. The absorbance was calculated from transmission T and reflection R for S-polarization at 8 degree incident angle as A = 1 – T – R. Optical density was calculated OD = - $\log_{10}$ A. Both T and R were measured by using PhotonRT

spectrometer. Oscillatory spectral dependence originates from the varying intensity of the light electric field inside the layer for various wavelengths.

[S1] V. Kanclíř, J. Václavík, and K. Žídek, Acta Phys. Pol. A **140**, 215–221 (2021).

[S2] R. Radoi, C. Gherasim, and M. Dinescu, M. J. Alloys Compd. **286**, 309-312 (1999).

[S3] A. Devos, and R. Côte. Physical Review B **70**, 125208 (2004).
